# Supplementary material for: The importance of motivation in selecting undergraduate medical students for extracurricular research programmes
Source: PLoS One. 2021 Nov 19;16(11):e0260193. doi: 10.1371/journal.pone.0260193 (PMC8604306; doi:10.1371/journal.pone.0260193)
Supplement: Attachment — Submitted filename: Reviewers comments.docx [file pone.0260193.s002.docx]

**Reviewer 1 comments:**

**The importance of motivation in selecting undergraduate medical students for extracurricular research programmes** (Manuscript Number PONE-D-21-11278R1)

This study attempts to throw light on the common oversight of an important benchmark in performance evaluation of medical researchers.

The paper is detailed, well explained and has incorporated good statistical tools. There a few observations made by the reviewer:

1. Kindly correct grammar inconsistencies at one or two areas in the paper (Eg. Inconsistencies with tenses: Line 273 – ‘All students were included’ is more appropriate as the study has been reported in the past tense throughout the paper)
2. If ERP drop-outs were more in the <7 group and this was statistically significant, one can argue that lack of motivation played an important role in the discontinuation from the ERP. Rather than dogmatically defending the findings with two contradictory theories for comparison of two different parameters between the same two study groups (viz. Reflected glory effect and Big fish in small pond theory), the acceptance to the paper can be enhanced with an open-mind approach that is:

- Authors can state in the paper that ‘the higher drop-out rate in <7 group could be justified by the big fish in small pond theory or could be attributed to the lack of motivation in the <7 group. Although the questionnaire shows no difference in motivation between the two groups, the finding of increased drop-outs confounds the result and warrants further research to investigate the reason for increased drop-outs in the <7 GPA group’
- If authors discuss that “Reflected glory effect” explains the increased self-perceived ability of the <7GPA group in the presence of >7 GPA group, authors should also mention that ‘from this perspective the presence of a >7 GPA group in the ERP is definitely a positive factor in the performance of <7 GPA group. However, the argument of the authors is not that GPA should not be a determinant factor of performance potential but the point here is that a GPA complemented with intrinsic motivation for research, research self-efficacy beliefs, perceptions of research and curiosity should be considered for selection of candidates for ERP in medical schools’.

If the authors accept the above-mentioned minor revision to the paper, the reviewer recommends that this paper be accepted for publication.
