# Supplementary material for: The importance of motivation in selecting undergraduate medical students for extracurricular research programmes
Source: PLoS One. 2021 Nov 19;16(11):e0260193. doi: 10.1371/journal.pone.0260193 (PMC8604306; doi:10.1371/journal.pone.0260193)
Supplement: Attachment — Submitted filename: Response to Reviewers.docx [file pone.0260193.s003.docx]

Dear Pathiyil Ravi Shankar,

We would like to thank you for the opportunity to revise our manuscript. In addition, we would like to thank the reviewers for the helpful review comments, which are greatly appreciated. The comments offer us guidance in the review process and help to improve our manuscript. Below we provide an overview on how we processed the review comments.

*Journal requirements*

*“Please review your reference list to ensure that it is complete and correct. If you have cited papers that have been retracted, please include the rationale for doing so in the manuscript text, or remove these references and replace them with relevant current references. Any changes to the reference list should be mentioned in the rebuttal letter that accompanies your revised manuscript. If you need to cite a retracted article, indicate the article’s retracted status in the References list and also include a citation and full reference for the retraction notice.”*
Done as requested.

*Reviewer 2*

*“The comments have been addressed and the project has to be taken forward as per suggestions to be more useful and relevant”*

We would like to thank the reviewer for checking our revised manuscript and are pleased to read that the comments have been addressed.

*Reviewer 3*

*“This study attempts to throw light on the common oversight of an important benchmark in performance evaluation of medical researchers. The paper is detailed, well explained and has incorporated good statistical tools.”*

We would like to thank the reviewer for reviewing our manuscript and for his/her positive words.

*1. Kindly correct grammar inconsistencies at one or two areas in the paper (Eg. Inconsistencies with tenses: Line 273 – ‘All students were included’ is more appropriate as the study has been reported in the past tense throughout the paper)*
Done as requested (revised manuscript lines 273, 315, and 318).

*“2. If ERP drop-outs were more in the <7 group and this was statistically significant, one can argue that lack of motivation played an important role in the discontinuation from the ERP. Rather than dogmatically defending the findings with two contradictory theories for comparison of two different parameters between the same two study groups (viz. Reflected glory effect and Big fish in small pond theory), the acceptance to the paper can be enhanced with an open-mind approach that is:
- Authors can state in the paper that ‘the higher drop-out rate in <7 group could be justified by the big fish in small pond theory or could be attributed to the lack of motivation in the <7 group. Although the questionnaire shows no difference in motivation between the two groups, the finding of increased drop-outs confounds the result and warrants further research to investigate the reason for increased drop-outs in the <7 GPA group’
- If authors discuss that “Reflected glory effect” explains the increased self-perceived ability of the <7GPA group in the presence of >7 GPA group, authors should also mention that* *‘from this perspective the presence of a >7 GPA group in the ERP is definitely a positive factor in the performance of <7 GPA group. However, the argument of the authors is not that GPA should not be a determinant factor of performance potential but the point here is that a GPA complemented with intrinsic motivation for research, research self-efficacy beliefs, perceptions of research and curiosity should be considered for selection of candidates for ERP in medical schools’.”*
The reviewer suggests that a lack of motivation played an important role in the discontinuation from the ERP and suggests that we should state in the paper that the higher drop-out rate could be justified by the big-fish-little-pond theory or could be attributed to the lack of motivation in the <7 group. We agree that the big-fish-little-pond theory is applicable here, which is why we mentioned this as a clarification in our discussion section. With regard to motivation, in general it could indeed be the case that motivation decreases and results in drop-out, however, we do not feel that it is justified to draw the conclusion from our data that a lack of motivation in the <7 group may cause the higher drop-out rate. When looking at the levels of intrinsic motivation at both timepoint 1 (table 1) and timepoint three (table 2) we can see that these levels are stable, or even marginally higher at timepoint three, implying that a lack of intrinsic motivation for research does not seem plausible. Especially as our non-inferiority approach shows that the <7 group is not inferior to the >7 group when it comes to intrinsic motivation for research. This view is corroborated by the coordinator of the ERP. We do believe that it could be that apparently motivation does not lead to finishing the programme, which we mention at page 18, line 364-366 of our revised manuscript (“it is remarkable that, though comparable in motivation for research, the drop-out in the <7 group is higher as compared to the ≥7 group, possibly implying that motivation does not lead to ERP completion”). Furthermore, the reviewer suggests that from the perspective of the reflective glory effect the presence of the ≥7 group is a positive factor for the performance of the <7 group, which we agree upon. Indeed, our argument is not that we should exclude the ≥7 group, rather that we should offer the <7 group the chance to participate within the ERP. In line with the reviewer’s suggestions, within the revised manuscript we now mention that ‘from this perspective, the presence of the ≥7 group is a positive factor for the performance of the <7 group’ (page 17, line 358-359).

Again, we would like to express our gratitude to the reviewers for the comments. We are very thankful for the time invested in our manuscript. We really feel it helped us to improve our manuscript considerably, and hope you acknowledge our improvements.

If any questions or concerns remain, please do not hesitate to contact us. Thank you for receiving our revised manuscript, we look forward to our collaboration on this manuscript.

On behalf of all authors,

Kind regards,

Belinda Ommering
