# Supplementary material for: The importance of motivation in selecting undergraduate medical students for extracurricular research programmes
Source: PLoS One. 2021 Nov 19;16(11):e0260193. doi: 10.1371/journal.pone.0260193 (PMC8604306; doi:10.1371/journal.pone.0260193)
Supplement: Attachment — Submitted filename: Response to Reviewers.docx [file pone.0260193.s004.docx]

Dear Pathiyil Ravi Shankar,

We would like to thank you for the opportunity to revise our manuscript. In addition, we would like to thank the reviewers for the helpful review comments, which are greatly appreciated. The comments offer us guidance in the review process and help to improve our manuscript. Below we provide an overview on how we processed the review comments.

*Journal requirements*

*“Please review your reference list to ensure that it is complete and correct. If you have cited papers that have been retracted, please include the rationale for doing so in the manuscript text, or remove these references and replace them with relevant current references. Any changes to the reference list should be mentioned in the rebuttal letter that accompanies your revised manuscript. If you need to cite a retracted article, indicate the article’s retracted status in the References list and also include a citation and full reference for the retraction notice.”*
Done as requested.

*Reviewer 3*

*“Reviewers concerns have been appropriately addressed; the manuscript may be accepted for publication in the revised format”*

We would like to thank the reviewer for checking our revised manuscript and are pleased to read that the reviewer suggests to accept the manuscript for publication.

*Reviewer 4*

*“The present manuscript is very interesting. Especially I strongly believe that lower academic curriculum should not be an "exclusion criteria" for further career.”*
We would like to thank the reviewer for reviewing our manuscript and for his positive words. We strongly agree with the reviewer that lower academic performance should not be an exclusion criteria.

*“Some issues. Introduction is well written but I think it should be shortened to the focus of this paper, that is the impact of "selection" of students for research”*
We agree with the reviewer that the focus of our paper is on selection of students for extracurricular research programmes (ERPs) and we are pleased to read that this came across as such. This study is situated in the context of a bigger problem that needs to be solved, i.e. the physician-scientist shortage, which is one of the reasons many ERPs were implemented. Therefore, we do feel that it is necessary to describe this problem and the reasons for implementing an ERP, logically following into the group that should be targeted to reach the desired outcome (i.e. more students pursuing a physician-scientist career). In this latter phase, questions regarding selection arise for which we investigated students with lower and higher academic performance within the ERP. We do feel, however, that we should keep the bigger picture in mind and provide this bigger picture to the broad readership as well. While still making sure that we did not lose the bigger picture and we did not go against previous reviewer requests to expand some parts within our introduction, we critically went through our manuscript and somewhat shortened the introduction at multiple points. We hope the editor understands our considerations and is satisfied with the current shortening of our introduction.

*“Methods: due to low sample size normal distribution should be assessed”*
We indeed assessed normal distribution at the start of our analysis phase. Data was normally distributed. For clarity purposes, we now mention that we established normal distribution of the data explicitly within the ‘analyses’ paragraph (page 11, line 242).

*“Methods/results: I think that income of the family of origin, and the level of education should be appraised as they impact of cv and opportunities”*

The reviewer suggests that income and level of education of the family of origin should be taken into account as they might impact cv and opportunities. We agree with the reviewer that these are factors that could certainly play a role in general performance, educational or career choices, and being selected for, for instance, an extracurricular research programme. However, we are afraid we do not have collected these data and are therefore not able to appraise them in the current study.

Again, we would like to express our gratitude to the reviewers for the comments. We are very thankful for the time invested in our manuscript. We really feel it helped us to improve our manuscript considerably, and hope you acknowledge our improvements.

If any questions or concerns remain, please do not hesitate to contact us. Thank you for receiving our revised manuscript, we look forward to our collaboration on this manuscript.

On behalf of all authors,

Kind regards,

Belinda Ommering
